# Physical Fitness, Selective Attention and Academic Performance in a Pre-Adolescent Sample

**DOI:** 10.3390/ijerph17176216

**Published:** 2020-08-27

**Authors:** José A. Páez-Maldonado, Rafael E. Reigal, Juan P. Morillo-Baro, Hernaldo Carrasco-Beltrán, Antonio Hernández-Mendo, Verónica Morales-Sánchez

**Affiliations:** 1 Pablo de Olavide University, Utrera Road, 41013 Sevilla, Spain; J.a.paezmaldonado@gmail.com; 2 Faculty of Psychology, University of Malaga, Teatinos Campus, 29071 Malaga, Spain; rafareigal@uma.es (R.E.R.); juanpablo.morillo@gmail.com (J.P.M.-B.); mendo@uma.es (A.H.-M.); 3 Playa Ancha University, Playa Ancha Avenue, 850 Valparaiso, Chile; hernaldo.carrasco@upla.cl

**Keywords:** physical fitness, selective attention, pre-adolescents, cognitive functioning

## Abstract

The purpose of the current study is to analyze the relationships between physical fitness, selective attention, and academic performance in pre-teens. The sample here consists of 135 participants between the ages of 10 and 12 (M = 11.05; SD *=* 0.61), with 39.26% female (*n* = 53) and 60.74% male (*n* = 82) participants. Horizontal and vertical jump distances, speed, and cardio-respiratory fitness were evaluated to assess physical fitness. The d2 Test of Attention was used to evaluate selective attention. In addition, data were obtained regarding participant academic performance by analyzing the academic performance. The results show significant relationships between the measures analyzed, highlighting positive associations between physical fitness, cognitive functioning, and academic performance. Thus, participants who were fitter scored better on tests of attention (*Z*_133_ = −4.07; *p* < 0.00007, Cohen’s *d* = 0.75, 95% CI (0.39, 1.11)) and concentration (*t*_133_ = −3.84; *p* < 0.0007, Cohen’s *d* = 0.69, 95% CI (0.33, 1.05)), as well as having higher academic performance (*Z*_133_ = −2.84; *p* < 0.0035, Cohen’s *d* = 0.39, 95% CI (0.04, 0.75)). Cardiorespiratory fitness was the measure of physical fitness that best explained these relationships. The results suggest that maintaining and improving the physical fitness of children and adolescents may help their brain function develop better.

## 1. Introduction

Recent research has shown that sedentary behaviors are widespread among children and adolescents, where individuals engage in less and less strenuous physical activity [1,2,3]. To change this situation, it would be appropriate to increase the level of physical activity through structured physical exercise, which can be achieved with specific physical training programs or by participating in sports [4]. Traditionally, the term physical activity refers to any motor behavior that requires energy expenditure, and this expenditure is usually not planned. When physical practice is planned and targeted, it is often called physical exercise instead [4]. In any case, for active behaviors to have a significant impact on health, it is desirable to engage in activities from moderate to high exercise intensity, which is currently not the case for a significant number of people at these age stages. This is alarming researchers who are increasingly observing the damage that these lifestyle habits can have on health [5]. Therefore, to ensure that physical activity with these characteristics is carried out, it would be convenient for children and adolescents to join planned and prolonged programs of physical exercise, which is known as physical training [6].

Among the researchers who have pointed out the relationship between the practice of physical activity or sport with the health of children and adolescents, in recent decades, those who have shown the repercussions that active lifestyles have on cognitive functioning stand out [7,8]. Thus, the importance of developing adequate physical exercise programs to favorably affect the central nervous system and improve cognitive functioning has been highlighted [6]. Thus, positive relationships between the practice of physical activity and various cognitive abilities, such as memory, executive functioning, attention, language processing, and processing speed, have been revealed at these ages [9,10,11,12]. These findings are very important at these ages, since correct cognitive functioning facilitates adaptation to the adolescent environment and contributes to better mental health and psychosocial development [13,14]. For example, motor competence has been found to be related to working memory in adolescents. Thus, adolescents with better motor development respond more appropriately in situations where this cognitive factor is essential for performance [15].

In previous research, techniques such as electroencephalography, functional near-infrared spectroscopy, functional magnetic resonance, positron emission tomography, and magnetoencephalography [16,17,18,19,20,21] have been applied in the field of sports science, and are allowing a better understanding of these types of phenomena. These neuroimaging techniques allow us to understand the effects of physical exercise on cognition more comprehensively, and it has been shown that exercising at higher exercise intensities leads to a more pronounced change in physical fitness and the brain [22,23]. As described in several previous studies, one of the most relevant indicators of improvements in cognitive function is the increase in aerobic capacity [24,25,26]. However, current studies have shown that it is necessary to continue investigating how the type of exercise affects cognitive functioning. Mora-Gonzalez et al. [27] observed that not only cardiorespiratory fitness, but also speed-agility positively affect various dimensions of cognitive functioning.

Among the investigations that have addressed the relationships between the practice of physical activity, condition, and cognitive functioning, some have focused on attention capacity [28,29,30] and specifically selective attention and concentration [26,31,32,33,34]. This kind of research is relevant due to the importance of this cognitive capacity in the adolescent stage, as it has a notable influence on multiple tasks performed by young people at these ages and their involvement in other, more complex cognitive functions [35,36]. In fact, selective attention is a skill that allows relevant stimuli to be addressed while ignoring other distractors or irrelevant information [37], which is essential for adolescent success in multiple academic or social tasks.

Several studies [26,34] have highlighted that adolescents who have a higher level of physical fitness show better attention and concentration abilities. In addition, aerobic capacity is the best predictor of cognitive functioning. Likewise, other studies, such as the one carried out by Cadenas-Sanchez et al. [38] regarding an adolescent sample, indicated that aerobic capacity and the degree of fatness was related to selective attention, with an existing interaction between both factors, showing that adolescents with a better profile of physical fitness and body composition were the ones with better scores in attention capacity. Although aerobic fitness has been shown to be the strongest predictor of selective attention and concentration, it has been observed that these cognitive factors are also associated with explosive strength performance and speed-agility performance [39]. In this regard, another study noticed that in adolescents, aerobic fitness, the percentage of body fat mass, and explosive strength performance were also correlated with performance in selective attention and concentration [40].

As some researchers have pointed out, the impact of physical exercise on cognitive functioning in children and adolescents can be transferred to everyday issues, such as academic performance. Álvarez-Bueno et al. [41] performed a systematic review and meta-analysis, in which they noted the existence of specific relationships between aerobic fitness and specific domains of academic achievement, such as language/reading or math-related skills. In a previous study, it was highlighted that an adequate school physical exercise program could improve the classroom behaviors of children and adolescents and could contribute to improving their academic performance [42]. This suggests that schools should consider that physical education classes could contribute to other academic disciplines, and thus are very powerful tools in the academic field. Furthermore, the findings of another previous meta-analysis indicated that long-term physical exercise programs were more effective than single bouts [43]. Therefore, school physical education departments could integrate this information when configuring school programs to favorably influence the general academic performance of students. However, other studies [44] investigating the influence of habitual physical activity levels on different academic subdomains showed that mathematical performance exhibited the strongest relationship with a habitual physical activity level.

As a summary of the previously mentioned studies, it could be concluded that there is considerable evidence linking cardiorespiratory fitness to attention and concentration in children, but the relationship between cardiorespiratory fitness and academic performance has not been sufficiently studied. For example, in another study, Reigal et al. [40] analyzed the relationships between physical fitness and cognitive functioning. However, they did not assess academic performance. Furthermore, the age range of the sample was from 14 to 15 years. To address this gap in the literature, the present study analyzes the relationships of some dimensions of physical fitness (e.g., cardiorespiratory fitness) with selective attention and concentration, as well as academic performance, in a pre-adolescent sample.

## 2. Materials and Methods

### 2.1. Participants

Here, 135 pre-adolescents (male gender, *n* = 82; female gender, *n* = 53) from the locality of La Roda de Andalucía (Seville), aged between 10 and 12 years (M ± SD = 11.05 ± 0.61) participated in this research study. The sampling was non-probabilistic and was selected from several educational centers. Age (between 10 and 12 years old) was considered an inclusion criterion. The exclusion criteria were the following: (a) Age less than 10 or more than 12 years old; (b) a physical injury that prevents physical fitness tests; (c) not having the informed consent form signed by the parents; (d) not performing the assessment tests following the correct protocol; or (e) having any neuropsychological problem that would prevent the cognitive assessment tests from being carried out correctly.

### 2.2. Instruments and Measures

Regarding selective attention and concentration, the d2 Test of Attention was used [45]. It consists of a total of 658 elements distributed in 14 rows. Individuals have 20 s to resolve each row and this is always done from left to right and from top to bottom. The items contain the letters “d” or “p” and may be accompanied by one or two stripes at the top, bottom, or both positions. The “d” must be crossed out with 2 stripes, regardless of the position. The following scores were found for each participant based on this test: TA (number of elements attempted), TH (number of hits), O (omissions or number of relevant stimuli not crossed out), C (omissions or errors), TOT (task effectiveness = TA − (O + C)), CON (concentration = TH − C), and VAR (index of variation between the last stimulus analyzed between different rows = (TA+) − (TA−), where TA+ would be the last stimulus analyzed in the row with the most attempted elements and TA− is the last stimulus analyzed in the row with the least attempted elements.

Participant height was assessed using a conventional measuring rod. Weight and percentage of fat mass were both determined using a bioimpedance meter (Tanita^®^ Body Composition Monitor model BC-601, Corp., Tokyo, Japan). A horizontal jump test was performed [46] and the vertical jump test was evaluated with an Apple application called “my jump” (Apple Inc., Cupertino, CA, USA) [47] to evaluate explosive strength in the lower limbs. Speed was also evaluated through a 30-m linear sprint test). This test was measured through the application “My Sprint” (Apple INC., Cupertino, CA, USA). This application has been recently validated [48]. The change of direction test was also performed through the V-cut test [49]. Cones, a tape measure, and a stopwatch (A164WA-1VES, Casio, Tokyo, Japan) were used for this test. In addition, maximum oxygen consumption was analyzed indirectly via the Course Navette test [50], using the formula VO2max = 31.025 + 3.238V − 3.248E + 0.1536VE (where V is the speed reached in the last completed stage and E is the age of the participant).

### 2.3. Procedure

The sample here was selected from various schools. First, permission was sought from the management of the center, and subsequently informed consent was obtained from the parents or legal guardians. In addition, the ethical principles of the Declaration of Helsinki [51] were respected and this study was approved by the Ethics Committee of the University of Malaga (no. 243, CEUMA Registry No. 18-2015-H).

Evaluations were conducted on two different days. First, anthropometric and body composition measurements were evaluated. Second, cognitive functioning tests were performed. In addition, average grades were obtained from the end-of-course transcript, which was used as a measure of academic performance.

### 2.4. Data Analysis

Descriptive and inferential analyses were performed. The Kolmogorov–Smirnov test was used to analyze the normality of the data. To determine the level of the relationship between the study variables, correlation analyses were performed. For this purpose, Pearson and Spearman correlations were used (±0.01 to ±0.19 = very weak correlation; ±0.20 to ±0.39 = weak correlation; ±0.40 to ±0.59 = moderate correlation; ±0.60 to ±0.79 = high correlation [52]). In addition, we aimed to investigate if the level of physical fitness could determine the measures of cognitive functioning and academic performance. Therefore, the predictive ability of the physical fitness measurements on selective attention and concentration, as well as on academic performance, were checked by regression analyses (successive steps). Finally, we tried to determine if the group with better physical fitness had better scores in cognitive functioning and academic performance. By means of cluster analysis (K-mean), two clusters were formed as a function of the physical fitness variables. Student’s *t*-test and Mann–Whitney U tests were used to assess differences in scores between clusters. In addition, the effect size was estimated by means of Cohen’s *d* [53]. The level of significance was set to α = 0.05. In addition, Bonferroni correction for multiple comparisons was performed to the minimize type I error. Here, SPSS version 20.0 (IBM Corp., Armonk, NY, USA) was used for statistical processing of the data.

## 3. Results

Table 1 shows the descriptive and normal statistics of the variables under study. As can be observed, the variables, commissions, and omissions of the d2 Test of Attention showed problems of normality.

The correlation analysis (Table 2) indicated significant and positive relationships between the variables of the horizontal jump and maximum oxygen consumption with various measures of the d2 Test of Attention. In addition, the speed test was significantly and negatively associated with some of the attention test measures. However, the omissions, commissions, and index of variation (d2 Test) were not related to any of the physical fitness variables.

The linear regression analyses (successive steps) can be seen in Table 3. The latest models generated for the total sample and those by gender are shown. Some variables were excluded as predictors due to a lack of significance (*p* > 0.05). The linearity assumptions were met in the relationship between predictor variables and criteria, homoscedasticity, and normal distribution of residuals. The Durbin–Watson values were between 1.78 and 2.03, so it can be assumed that the residuals are independent and the assumption of the independence of the independent variables with respect to the dependent one is fulfilled [54].

Through cluster analysis (K-means) two clusters were generated depending on the variables of the percentage of body fat mass, speed test, V-cut test, and VO2max. Each case was well classified, since the maximum distance of each one from the center of its group (14.24) was less than the distance between the centers of the clusters (26.58). The two groups constituted were characterized by a higher percentage of fat mass and worse physical performance (group 1) (*n* = 49; 23 boys and 26 girls) or a lower percentage of fat mass and better physical performance (group 2) (*n* = 86; 59 boys and 27 girls).

Table 4 shows the descriptive and normal statistics of the variables analyzed according to the physical fitness measurements. The analyses performed (Student’s *t*-test and Mann–Whitney U test, with Bonferroni correction) indicated statistically significant differences (the *p*-values after Bonferroni correction were as follows: *p* < 0.0035, *p* < 0.0007, and *p* < 0.00007) between the two groups in the measurements of physical fitness regarding the percentage of fat mass (*Z*_133_ = −9.60; *p* < 0.00007, Cohen’s *d* = −3.38, 95% CI (−3.92, −2.85)), speed test (*t*_133_ = 3.72; *p* < 0.0007, Cohen’s *d* = −0.67, 95% CI (−1.03, −0.31)), change of direction test (*t*_133_ = 3.26; *p* < 0.0035, Cohen’s *d* = −0.59, 95% CI (−0.94, −0.23)), and maximum oxygen consumption (*Z*_133_ = −4.86; *p* < 0.00007, Cohen’s *d* = 0.69, 95% CI (0.33, 1.05)). Significant differences were also observed in the d2 Test measures regarding the TA measures (*Z*_133_ = −4.33; *p* < 0.00007, Cohen’s *d* = 0.79, 95% CI (0.42, 1.15)), TH (*Z*_133_ = −4.08; *p* < 0.00007, Cohen’s *d* = 0.78, 95% CI (0.42, 1.14)), TOT (*Z*_133_ = −4.07; *p* < 0.00007, Cohen’s *d* = 0.75, 95% CI (0.39, 1.11)), CON (*t*_133_ = −3.84; *p* < 0.0007, Cohen’s *d* = 0.69, 95% CI (0.33, 1.05)), and VAR (*Z*_133_ = −3.73; *p* < 0.0007, Cohen’s *d* = 0.67, 95% CI (0.31, 1.03)), as well as the academic performance values (*Z*_133_ = −2.84; *p* < 0.0035, Cohen’s *d* = 0.39, 95% CI (0.04, 0.75)).

## 4. Discussion

The purpose of this paper was to determine the relationships between physical fitness, cognitive functioning, and academic performance in a group of pre-teens. For this purpose, correlation and linear regression analyses have been carried out. In addition, groups of participants have been generated based on measures of physical fitness and body composition and scores have been compared between them. Thus, two conglomerates were generated characterized by a high percentage of fat mass and a lower physical performance, or by a lower percentage of fat mass and a better physical performance. In general, it can be observed that there is a positive relationship between physical performance with cognitive functioning measures and academic performance, as well as a negative relationship between the percentage of fat mass with selective attention and concentration. These results reveal statistically significant relationships between the study variables and contribute to expanding the scientific evidence that has previously indicated the relationship between physical fitness and cognitive functioning at these ages [7,55].

In particular, our results show that pre-teens who scored better on the tests of speed, change of direction, and cardiorespiratory fitness exhibited a superior performance in terms of their selective attention and concentration. This is in accordance with previous studies revealing a positive association between physical fitness and cognitive functioning, especially in the cognitive domains of attention and concentration [26,31,34,38]. In addition, cardiorespiratory fitness operationalized via indirect maximal oxygen consumption is the variable that explained the largest variance in the relationship between physical fitness and cognitive functioning. This phenomenon is in line with previous studies showing that cardiorespiratory fitness is associated with the level of cognitive functioning in adolescents [24,25,26]. As described in previous studies, the development of cardio-respiratory fitness is associated with a greater hippocampal or basal ganglia volume [56]. In addition, in other studies, it has been proposed that greater aerobic fitness increases the microstructure of white matter, which increases the efficiency of communication between brain regions [57].

However, more research is needed to determine how other measures of physical fitness affect cognitive functioning in children and adolescents. Although cardiorespiratory fitness is more strongly related to cognitive functioning, this study reveals how other dimensions such as speed and speed-agility have been associated with selective attention and concentration. This issue has been highlighted in previous studies that have investigated how different dimensions of physical fitness are related to cognitive functions [39,40]. For example, physical exercise programs that combine cardiorespiratory fitness and speed-agility have previously been proposed to improve cognitive functioning [27]. Thus, Esteban-Cornejo et al. [58] highlighted that these programs have a positive impact on the development of different brain areas, such as the premotor cortex, supplementary motor cortex, hippocampus, caudate, inferior temporal and frontal gyri, superior temporal gyrus, parahippocampal gyrus, and calcarine cortex. Understanding the influence of different fitness dimensions (e.g., cardiorespiratory fitness, muscular fitness, motor-cognitive fitness) on cognitive function and academic performance is essential to enhance the understanding of the interaction between exercise and cognition, and, in turn, to better tailor physical exercise programs to the needs of the individual.

Likewise, the cluster analyses carried out here have highlighted significant differences between the two groups. In this context, children with a relatively high fitness level exhibited superior cognitive performance as compared to children with a relatively low fitness level. Hence, our findings are in accordance with the previous literature suggesting that a superior physical fitness level is linked to better cognitive functioning in children and adolescents. Along these lines, there are notable differences between the two groups, suggesting that this is a fact to be taken into account and that it could be relevant to the integral development of children and adolescents. Thus, Cadenas-Sanchez et al. [38] pointed out the interactions that could be established between poor physical performance and an inadequate body composition profile. In this paper, linear regression analyses have not included the percentage of body fat mass, but the conglomerate with the best scores has been characterized by a better physical fitness and a lower percentage of body fat mass. This fact, which will have to be further addressed in future investigations, could indicate the need to monitor the two parameters for a better prediction of health in general and cognitive functioning, particularly for children and adolescents.

Furthermore, it has been highlighted that possessing good cognitive functioning at these ages is related to better psychosocial adjustment for children and adolescents, better adaptation to the environment, and greater success in daily activities [13,14]. For example, academic activities are one area that could benefit, with an important impact at this age. In this sense, it has been observed in the present research that better physical fitness is not only associated with cognitive functioning, but also with academic performance. This has already been highlighted previously [59,60,61], and its impact on the development processes of children and adolescents should continue to be an interesting line of study. Although specific competencies have not been analyzed in the current research, and only a general index of academic performance has been analyzed, the data suggest that an improved physical condition could have an impact on certain intellectual abilities that generate better functioning in different academic areas. However, this phenomenon cannot be deduced from the findings obtained here and will have to be explored in future research.

In summary, this study highlights the important role of physical fitness when analyzing factors associated with health or well-being in children. Therefore, when assessing the lifestyles of children and adolescents, it should not only be pointed out that they are healthy or that they promote active habits, but it is also necessary to contribute to improving the level of physical fitness so that the effects are more forceful. Thus, as suggested by authors such as Chaddock et al. [62], Esteban-Cornejo et al. [58], or Reloba-Martínez et al. [26], when physical activity is carried out, it must overcome a certain threshold of a mismatch of supply and demand that allows the organism to adapt to it, and this process generates a sufficient impact on the brain for its functioning to be optimized.

This research has a number of limitations. For example, the design used here does not allow causal relationships to be established. Therefore, in future research, others studies of a longitudinal or quasi-experimental type could be implemented to facilitate this type of interpretation. Furthermore, even though our sample size is too small to investigate the influence of gender, this aspect should be considered in future investigations, as there is some evidence suggesting gender-specific effects of physical exercise on cognitive performance [2,3,4]. Moreover, we also recommend that future studies consider variables such as study time or rest, as well as feeding or the use of new technologies, which might influence the associations between physical fitness, cognitive performance, and academic achievement. Another possible limitation is the indirect assessment of maximal oxygen consumption, which might have generated some measurement error. Additionally, only specific physical fitness dimensions have been assessed. Future research, therefore, should consider an assessment of different dimensions of fitness (e.g., cardiorespiratory fitness, muscular fitness, motor-cognitive fitness), which could provide a more comprehensive insight into the relationships between specific physical fitness dimensions, cognitive functioning, and academic performance.

## 5. Conclusions

The results found here provide evidence of the connections between physical fitness, cognitive functioning, and academic performance. Specifically, relationships have been found between speed, speed-agility, and cardiorespiratory fitness with selective fitness and concentration, as well as between cardiorespiratory fitness and academic performance. In addition, cardiorespiratory fitness is the best predictor of the measures of cognitive functioning and academic performance. This suggests that regular physical exercise, which normally increases physical fitness levels, should be promoted among children, adolescents, and their parents in order to foster health and integral development. Thus, those responsible for public and private organizations, schools, and sports clubs should focus on creating active lifestyles and facilitating access to physical practice at these ages.

## Figures and Tables

**Table 1:** Descriptive measurements and Kolmogorov–Smirnov test for the analyzed variables.

Study Variables	M	SD	S	K	K–S
% Body fat mass	16.07	6.41	0.45	−0.62	1.09
Sprint 30 m (sg)	5.48	0.45	−0.36	0.02	1.33
Vertical jump (cm)	35.88	6.46	0.00	−1.20	1.17
Horizontal jump (cm)	129.80	12.07	0.67	−0.14	1.24
V-cut (sg)	8.17	1.11	0.37	−1.02	1.30
VO2max (mL/kg/min)	39.04	4.65	0.59	−0.11	1.17
D2-TA	62.41	14.62	−0.02	−1.11	1.21
D2-TH	61.86	14.19	0.08	−1.18	1.25
D2-O	65.99	11.19	−0.36	−0.58	1.97 ***
D2-C	51.11	14.02	0.15	−1.23	2.64 ***
D2-TOT	64.35	14.56	0.01	−1.04	1.32
D2-CON	61.63	13.21	0.11	−0.97	1.08
D2-VAR	59.67	13.60	0.15	−0.95	1.32
SAR (1–10)	7.59	1.38	−0.58	−0.49	1.20

S = skewness; K= kurtosis; K–S = Kolmogorov–Smirnov value; VO2max = maximum oxygen consumption (mL/kg/min); TA = total number of attempts; TH = total hits; O = omissions; C = commissions; TOT = total effectiveness in the test; CON = concentration index; VAR = variation index; SAR = school average rating. *** *p* < 0.001.

**Table 2:** Analysis of correlations between study variables.

Study Variables	D2-TA	D2-TH	D2-O	D2-C	D2-TOT	D2-CON	D2-VAR	SAR
% Body fat mass	−0.27 **	−0.26 **	−0.07	0.03	0.26 **	−0.21 *	−0.22 *	−0.09
Sprint 30 m (sg)	−0.28 **	−0.27 **	−0.08	0.06	0.24 **	−0.26 **	−0.23 **	−0.11
Vertical jump (cm)	0.04	0.05	0.04	−0.06	0.11	0.02	0.03	−0.04
Horizontal jump (cm)	0.06	0.07	−0.05	0.01	0.10	0.04	0.06	0.05
V-cut (sg)	−0.31 ***	−0.30 ***	−0.11	−0.06	−0.29 ***	−0.28 **	−0.26 **	−0.15
VO2max (mL/kg/min)	0.44 ***	0.42 ***	0.16	−0.07	0.41 ***	0.40 ***	0.35 ***	0.18 *

VO2max = maximum oxygen consumption (mL/kg/min); TA = total number of attempts; TH = total hits; O = omissions; C = commissions; TOT = total effectiveness in the test; CON = concentration index; VAR = variation index; SAR = school average rating. * *p* < 0.05; ** *p* < 0.01; *** *p* < 0.001.

**Table 3:** Linear regression analysis (successive steps).

Criterion	ANOVA	R	R2 Adjusted	D–W	Predictors	Beta	t	T	VIF
D2-TA	31.28 ***	0.44	0.18	1.78	VO2max	0.44	5.59 ***	1.00	1.00
D2-TH	28.35 ***	0.42	0.17	1.96	VO2max	0.42	5.33 ***	1.00	1.00
D2-TOT	33.55 ***	0.45	0.20	2.01	VO2max	0.45	5.79 ***	1.00	1.00
D2-CON	28.98 ***	0.42	0.17	2.03	VO2max	0.42	5.38 ***	1.00	1.00
D2-VAR	23.54 ***	0.39	0.15	2.01	VO2max	0.39	4.85 ***	1.00	1.00
SAR	5.04 *	0.19	0.03	1.82	VO2max	0.19	2.25 *	1.00	1.00

VO2max = maximum oxygen consumption; TA = total number of attempts; TH = total hits; TOT = total test effectiveness; CON = concentration index; VAR = variation index; SAR = school average rating; D–W = Durbin–Watson value; T = tolerance; IVF = variance inflation factor. * *p* < 0.05; *** *p* < 0.001.

**Table 4:** Descriptive and normal measures of the variables analyzed as a function of physical fitness.

Study Variables	Group 1					Group 2				
	M	SD	S	K	K–S	M	SD	S	K	K–S
% Body fat mass	23.29	3.48	0.92	1.85	0.85	11.95	3.28	0.05	−1.19	1.46 *
Sprint 30 m (sg)	5.66	0.42	−0.62	0.75	0.79	5.37	0.44	−0.27	0.04	1.07
Vertical jump (cm)	34.92	4.84	−0.01	−0.50	0.60	36.43	7.19	−0.12	−1.47	1.22
Horizontal jump (cm)	126.63	9.95	0.49	0.04	0.74	131.60	12.83	0.60	−0.51	1.18
V-cut (sg)	8.57	1.19	−0.05	−1.48	1.19	7.94	1.00	0.55	−0.48	1.05
VO2max (mL/kg/min)	37.08	4.81	1.54	2.17	1.40 *	40.15	4.19	0.28	−0.15	1.12
D2-TA	55.53	12.95	0.93	0.54	1.12	66.33	14.12	−0.53	−0.71	1.47 *
D2-TH	55.24	12.07	1.13	1.03	1.29	65.63	13.98	−0.44	−0.94	1.55 *
D2-O	64.35	11.65	−0.09	−0.77	1.18	66.92	10.88	−0.52	−0.30	1.58 *
D2-C	50.82	13.07	0.10	−1.11	1.53 *	51.28	14.61	0.17	−1.31	2.14 ***
D2-TOT	57.76	12.37	0.90	0.64	1.14	68.10	14.45	−0.47	−0.72	1.55 *
D2-CON	56.12	11.67	0.81	0.33	1.04	64.77	13.07	−0.27	−0.83	1.28
D2-VAR	54.12	12.23	1.01	0.61	1.01	62.83	13.39	−0.27	−0.77	1.67 **
SAR (1–10)	7.23	1.27	−0.41	0.14	1.26	7.76	1.40	−0.74	−0.56	1.48 *

S = skewness; K = kurtosis; K–S = Kolmogorov–Smirnov; VO2max = maximum oxygen consumption (mL/kg/min); TA = total number of attempts; TH = total hits; O = omissions; C = commissions; TOT = total effectiveness in the test; CON = concentration index; VAR = variation index; SAR = school average rating. * *p* < 0.05; ** *p* < 0.01; *** *p* < 0.001.

## References

[1] Ekelund U., Luan J., Sherar L.B., Esliger D.W., Griew P., Cooper A. (2012). Moderate to vigorous physical activity and sedentary time and cardiometabolic risk factors in children and adolescents. J. Am. Med. Assoc..

[2] Morton K.L., Atkin A.J., Corder K., Suhrcke M., Van Sluijs E.M.F. (2016). The school environment and adolescent physical activity and sedentary behaviour: A mixed-studies systematic review. Obes. Rev..

[3] Zylke J., Bauchner H. (2016). The unrelenting challenge of obesity. J. Am. Med. Assoc..

[4] Herold F., Müller P., Gronwald T., Müller N.G. (2019). Dose-response matters!—A perspective on the exercise prescription in exercise-cognition research. Front. Psychol..

[5] Hayes G., Dowd K.P., MacDonncha C., Donnelly A.E. (2019). Tracking of physical activity and sedentary behavior from adolescence to young adulthood: A systematic literature review. J. Adolesc. Health.

[6] Herold F., Törpel A., Hamacher D., Budde H., Gronwald T. (2020). A discussion on different approaches for prescribing physical interventions—Four roads lead to Rome, but which one should we choose?. J. Pers. Med..

[7] Westfall D.R., Gejl A.K., Tarp J., Wedderkopp N., Kramer A.F., Hillman C.H., Bugge A. (2018). Associations between aerobic fitness and cognitive control in adolescents. Front. Psychol..

[8] Xue Y., Yang Y., Huang T. (2019). Effects of chronic exercise interventions on executive function among children and adolescents: A systematic review with meta-analysis. Br. J. Sports Med..

[9] Donnelly J.E., Hillman C.H., Castelli D., Etnier J.L., Lee S., Tomporowski P.D., Szabo-Reed A.N. (2016). Physical activity, fitness, cognitive function, and academic achievement in children: A systematic review. Med. Sci. Sports Exerc..

[10] Li J.W., O’Connor H., O’Dwyer N., Orr R. (2017). The effect of acute and chronic exercise on cognitive function and academic performance in adolescents: A systematic review. J. Sci. Med. Sport.

[11] Scudder M.R., Federmeier K.D., Raine L.B., Direito A., Boyd J.K. (2014). The association between aerobic fitness and language processing in children: Implications for academic achievement. Brain Cogn..

[12] Verburgh L., Königs M., Scherder E.J., Oosterlaan J. (2014). Physical exercise and executive functions in preadolescent children, adolescents and young adults: A meta-analysis. Br. J. Sports Med..

[13] Gale C.R., Cooper R., Craig L., Elliott J., Kuh D., Richards M., Deary I.J. (2012). Cognitive function in childhood and lifetime cognitive change in relation to mental wellbeing in four cohorts of older people. PLoS ONE.

[14] Lubans D., Richards J., Hillman C., Faulkner G., Beauchamp M.N., Kelly P., Smith J., Raine L., Biddle S. (2016). Physical Activity for Cognitive and Mental Health in Youth: A Systematic Review of Mechanisms. Pediatrics.

[15] Ludyga S., Herrmann C., Mücke M., Andrä C., Brand S., Pühse U., Gerber M. (2018). Contingent negative variation and working memory maintenance in adolescents with low and high motor competencies. Neural Plast..

[16] Boecker H., Drzezga A. (2016). A perspective on the future role of brain pet imaging in exercise science. Neuroimage.

[17] Chen A.G., Zhu L.N., Yan J., Yin H.C. (2016). Neural basis of working memory enhancement after acute aerobic exercise: fMRI study of preadolescent children. Front. Psychol..

[18] Gutmann B., Zimmer P., Hülsdünker T., Lefebvre J., Binnebößel S., Oberste M., Bloch W., Strüder H.K., Mierau A. (2018). The effects of exercise intensity and post-exercise recovery time on cortical activation as revealed by EEG alpha peak requency. Neurosci. Lett..

[19] Huang P., Fang R., Li B.Y., Chen S.D. (2016). Exercise-related changes of networks in aging and mild cognitive impairment brain. Front. Aging Neurosci..

[20] Herold F., Wiegel P., Scholkmann F., Müller N.G. (2018). Applications of functional near-infrared spectroscopy (fnirs) neuroimaging in exercise-cognition science: A systematic, methodology-focused review. J. Clin. Med..

[21] Lloyd-Fox S., Blasi A., Elwell C.E. (2010). Illuminating the developing brain: The past, present and future of functional near infrared spectroscopy. Neurosci. Biobehav. Rev..

[22] Jonasson L.S., Nyberg L., Kramer A.F., Lundquist A., Riklund K., Boraxbekk C.J. (2017). Aerobic exercise intervention, cognitive performance, and brain structure: Results from the physical influences on brain in aging (PHIBRA) study. Front. Aging Neurosci..

[23] Schwarb H., Johnson C.L., Daugherty A.M., Hillman C.H., Kramer A.F., Cohen N.J., Barbey A.K. (2017). Aerobic fitness, hippocampal viscoelasticity, and relational memory performance. Neuroimage.

[24] Pontifex M.M., Colby J.B., Sowell E.R., Nagel B.J. (2014). White matter connectivity and aerobic fitness in male adolescents. Dev. Cogn. Neurosci..

[25] Pontifex M.B., Raine L.B., Johnson C.R., Chaddock L., Voss M.W., Cohen N.J., Kramer A.F., Hillman C.H. (2011). Cardiorespiratory fitness and the flexible modulation of cognitive control in preadolescent children. J. Cogn. Neurosci..

[26] Reloba-Martínez S., Reigal-Garrido R.E., Hernández-Mendo A., Martínez-López E.J., Martín-Tamayo I., Chirosa-Ríos L.J. (2017). Effects of vigorous extracurricular physical exercise on the attention of schoolchildren. Rev. Psicol. Deporte.

[27] Mora-Gonzalez J., Esteban-Cornejo I., Solis-Urra P., Migueles J.H., Cadenas-Sanchez C., Molina-Garcia P., Rodriguez-Ayllon M., Hillman C.H., Catena A., Pontifex M.B. (2020). Fitness, physical activity, sedentary time, inhibitory control, and neuroelectric activity in children with overweight or obesity: The ActiveBrains project. Psychophysiology.

[28] Khan N.A., Hillman C.H. (2014). The relation of childhood physical activity and aerobic fitness to brain function and cognition: A review. Pediatr. Exerc. Sci..

[29] Verret C., Guay M.C., Berthiaume C., Gardiner P., Béliveau L. (2012). A physical activity program improves behavior and cognitive functions in children with ADHD: An exploratory study. J. Atten. Disord..

[30] Vanhelst J., Béghin L., Duhamel A., Manios Y., Molnar D., De Henauw S., Moreno L.A., Ortega F.B., Sjöström M., Widhalm K. (2016). Physical activity is associated with attention capacity in adolescents. J. Pediatr..

[31] Cserjési R., Molnár D., Luminet O., Lénárd L. (2007). Is there any relationship between obesity and mental flexibility in children?. Appetite.

[32] de Sousa A.F.M., Medeiros A.R., Del Rosso S., Stults-Kolehmainen M., Boullosa D.A. (2019). The influence of exercise and physical fitness status on attention: A systematic review. Int. Rev. Sport. Exerc. Psychol..

[33] Hillman C.H., Kamijo K., Scudder M. (2011). A review of chronic and acute physical activity participation on neuroelectric measures of brain health and cognition during childhood. Prev. Med..

[34] Pérez-Lobato R., Reigal R.E., Hernández-Mendo A. (2016). Relationships between physical practice, physical condition, and attention in a sample of adolescents. Rev. Psicol. Deporte.

[35] Perlman S.B., Hein T.C., Stepp S.D. (2014). Emotional reactivity and its impact on neural circuitry for attention–emotion interaction in childhood and adolescence. Dev. Cogn. Neurosci..

[36] Rabiner D.L., Godwin J., Dodge K.A. (2016). Predicting academic achievement and attainment: The contribution of early academic skills, attention difficulties, and social competence. Sch. Psychol. Rev..

[37] Giuliano R.J., Karns C.M., Neville H.J., Hillyard S.A. (2014). Early auditory evoked potential is modulated by selective attention and related to individual differences in visual working memory capacity. J. Cogn. Neurosci..

[38] Cadenas-Sanchez C., Vanhelst J., Ruiz J.R., Castillo-Gualda R., Libuda L., Labayen I., De Miguel-Etayo P., Marcos A., Molnár E., Catena A. (2017). Fitness and fatness in relation with attention capacity in European adolescents: The HELENA study. J. Sci. Med. Sport.

[39] Reigal R.E., Moral-Campillo L., Juárez-Ruiz de Mier R., Morillo-Baro J.P., Morales-Sánchez V., Pastrana J.L., Hernández-Mendo A. (2020). Physical fitness level is related to attention and concentration in adolescents. Front. Psychol..

[40] Reigal R.E., Moral-Campillo L., Morillo-Baro J.P., Juárez-Ruiz de Mier R., Hernández-Mendo A., Morales-Sánchez V. (2020). Physical exercise, fitness, cognitive functioning, and psychosocial variables in an adolescent sample. Int. J. Environ. Res. Public Health.

[41] Álvarez-Bueno C., Hillman C.H., Cavero-Redondo I., Sánchez-López M., Pozuelo-Carrascosa D.P., Martínez-Vizcaíno V. (2020). Aerobic fitness and academic achievement: A systematic review and meta-analysis. J. Sports Sci..

[42] Álvarez-Bueno C., Pesce C., Cavero-Redondo I., Sánchez-López M., Garrido-Miguel M., Martínez-Vizcaíno V. (2017). Academic achievement and physical activity: A meta-analysis. Pediatrics.

[43] De Greeff J.W., Bosker R.J., Oosterlaan J., Visscher C., Hartman E. (2017). Effects of physical activity on executive functions, attention and academic performance in preadolescent children: A meta-analysis. J. Sci. Med. Sport.

[44] Singh A.S., Saliasi E., van den Berg V., Uijtdewilligen L., de Groot R.H.M., Jolles J., Andersen L.B., Bailey R., Chang Y.-K., Diamond A. (2018). Effects of physical activity interventions on cognitive and academic performance in children and adolescents: A novel combination of a systematic review and recommendations from an expert panel. Br. J. Sports Med..

[45] Brickenkamp R. (2001). D-2. Attention Task, Madrid.

[46] Eurofit (1993). Handbook for the Eurofit Test on Physical Fitness.

[47] Balsalobre-Fernández C., Glaister M., Lockey R.A. (2015). The validity and reliability of an iPhone app for measuring vertical jump performance. J. Sports Sci..

[48] Romero-Franco N. (2017). Sprint performance and mechanical outputs computed with an iPhone app: Comparison with existing reference methods. Eur. J. Sport Sci..

[49] Gonzalo-Skok O., Tous-Fajardo J., Suarez-Arrones L., Arjol-Serrano J.L., Casajús J.A., Mendez-Villanueva A. (2015). Validity of the V-cut test for young basketball players. Int. J. Sports Med..

[50] Léger L.A., Mercier D., Gadoury C., Lambert J. (1988). The multistage 20 metre shuttle run test for aerobic fitness. J. Sports Sci..

[51] World Medical Association (2013). World Medical Association Declaration of Helsinki: Ethical principles for medical research involving human subjects. J. Am. Med. Assoc..

[52] Evans J.D. (1996). Straightforward Statistics for the Behavioral Sciences.

[53] Cohen J. (1998). Statistical Power Analysis for the Behavioural Sciences.

[54] Pardo A., Ruiz M.A. (2005). Data Analysis with SPSS 13 Base.

[55] Kao S.C., Drollette E.S., Scudder M.R., Raine L.B., Westfall D.R., Pontifex M.B., Hillman C.H. (2017). Aerobic fitness is associated with cognitive control strategy in preadolescent children. J. Motor Behav..

[56] Chaddock L., Erickson K.I., Prakash R.S., VanPatter M., Voss M.W., Pontifex M.B., Raine L.B., Hillman C.H., Kramer A.F. (2010). Basal ganglia volume is associated with aerobic fitness in preadolescent children. Dev. Neurosci..

[57] Chaddock-Heyman L., Erickson K.I., Holtrop J.L., Voss M.W., Pontifex M.B., Raine L.B., Hillman C.H., Kramer A.F. (2014). Aerobic fitness is associated with greater white matter integrity in children. Front. Hum. Neurosci..

[58] Esteban-Cornejo I., Cadenas-Sanchez C., Contreras-Rodriguez O., Verdejo-Roman J., Mora-Gonzalez J., Migueles J.H., Henriksson P., Davis C.L., Verdejo-García A., Catena A. (2017). A whole brain volumetric approach in overweight/obese children: Examining the association with different physical fitness components and academic performance. The ActiveBrains project. Neuroimage.

[59] Fernandes V.R., Ribeiro M.L.S., Melo T., de Tarso Maciel-Pinheiro P., Guimarães T.T., Araújo N.B., Ribeiro S., Deslandes A.C. (2016). Motor coordination correlates with academic achievement and cognitive function in children. Front. Psychol..

[60] Santana C.C.A., Azevedo L.B., Cattuzzo M.T., Hill J.O., Andrade L.P., Prado W.L. (2017). Physical fitness and academic performance in youth: A systematic review. Scand. J. Med. Sci. Sports.

[61] Chaddock-Heyman L., Erickson K.I., Kienzler C., King M., Pontifex M.B., Raine L.B., Hillman C.H., Kramer A.F. (2015). The role of aerobic fitness in cortical thickness and mathematics achievement in preadolescent children. PLoS ONE.

[62] Chaddock-Heyman L., Erickson K.I., Chappell M.A., Johnson C.L., Kienzler C., Knecht A., Drollette E.S., Raine L.B., Scudder M.R., Kao S.C. (2016). Aerobic fitness is associated with greater hippocampal cerebral blood flow in children. Dev. Cogn. Neurosci..

